# Endoscopic Spinal Decompression: A Retrospective Review of Pain Outcomes at an Academic Medical Center

**DOI:** 10.7759/cureus.19112

**Published:** 2021-10-29

**Authors:** Geoffrey D Panjeton, Holden L Brown, Sam Searcy, Matthew Meroney, Sanjeev Kumar

**Affiliations:** 1 Anesthesiology, University of Florida College of Medicine, Gainesville, USA

**Keywords:** minimally invasive spine surgery, interventional pain, disc herniation, lumbar spinal stenosis, endoscopic spine decompression

## Abstract

Introduction: Spinal stenosis is a chronic, debilitating condition that is expected to affect an increasing number of people as the population ages. Symptomatic spinal stenosis, like other spine pathologies, including disc herniation and degenerative disc disease, traditionally required an open decompressive surgical approach if more conservative approaches failed. An emerging alternative has been developed to address the needs of this population of patients in the form of endoscopic spine surgery (ESS). Advantages of ESS include minimal tissue trauma, decreased risk of damage to the neurovascular structures, minimal epidural fibrosis/scarring, reduced hospital stay, early functional recovery, and improved cosmetic outcomes. The purpose of this study was to review the outcomes of patients undergoing transforaminal endoscopic spinal decompression at an academic pain program.

Methods: We conducted a retrospective review of electronic medical records with approval from the University of Florida Institutional Review Board (IRB #202001529). Twenty patients underwent successful transforaminal endoscopic lumbar spinal decompression surgery at UF Health Pain Medicine from July 1, 2019, to June 1, 2020. The majority of cases were performed at L4-5 (n = 14), followed by an equal number (n = 3) of cases at L3-4 and L5-S1. Preoperative and postoperative visual analog scale (VAS) pain scores from patients' pain clinic appointments were obtained from the electronic health records system to assess the intervention as a pain relief strategy.

Results: Patients had an average pain reduction of 82% (SD = 31%), resulting in an average postoperative pain score of 1.8 (SD = 2.8) on a 10-point VAS.

Conclusion: This study highlights the benefits of endoscopic spine surgery for patients, including pain reduction and reduced scarring.

## Introduction

Spinal stenosis is a chronic, debilitating condition that affects up to 9.3% of people, most commonly in the sixth or seventh decade of life [1]. The number of people affected is expected to increase with an aging population worldwide and is predicted to reach 64 million within the decade in our country [2]. These patients are often referred to interventional pain specialists, with symptoms concerning neurogenic claudication. Interventional pain specialists may use a variety of interventions to help treat the pain, including epidural steroid injections, percutaneous image-guided minimally invasive lumbar decompression, or interspinous spacer placement. If these interventions are unable to provide durable relief, these patients are typically given the option for referral to spine surgery or conservative management with medications and physical therapy. Surgical intervention for spinal stenosis traditionally includes decompression by soft tissues, and intervertebral disc dissection/debulking, and surgical removal of bony structures. In the long term, patients may develop facet joint and spine destabilization, requiring more surgeries, and there could be a protracted recovery process. An emerging alternative developed to address this population is endoscopic spine surgery. Advantages of ESS include minimal tissue trauma, decreased risk of damage to the neural structures and epidural vessels with a decrease in subsequent epidural fibrosis/scarring, reduced hospital stay, early functional recovery, and improved cosmetic outcomes [3,4].

Potential complications of this procedure include surgical site infection, epidural abscess, epidural hematoma, posterior dural tear, ventral dural tear, and nerve root injury. Ventral dural tears are particularly feared because they are very difficult to repair, and larger tears can lead to herniation of the nerve root(s) through the tear with potential strangulation of the nerve root, leading to catastrophic consequences. These complications are avoided by maintaining an adequate view of target structures throughout and use of safe landmarks [3]. Training in ESS is critical before attempting this procedure because there is a steep learning curve for spine endoscopy. Complication avoidance, recognition of intraoperative pitfalls, and training in complication management and mitigation should be a critical part of the spine endoscopy training. Additionally, access to spine surgery colleagues for referral and admitting privileges at a hospital are recommended to manage potential major complications.

The evidence supporting ESS as an alternative to more invasive surgical options is steadily growing. However, there is a dearth of literature reviewing the outcomes of interventional pain physicians using ESS. The purpose of this study was to review the outcomes of patients undergoing endoscopic spinal decompression from an academic pain program.

## Materials and methods

Study design

After the University of Florida Institutional Review Board approval (IRB#202001529), we performed a retrospective electronic medical record review of 22 patients undergoing transforaminal endoscopic spine decompression between July 1, 2019, and June 1, 2020, by UF Health Pain Medicine physicians. Data were extracted from electronic medical records from the Epic electronic health record by three members of the study staff who underwent training to standardize data collection methods. Two patients were excluded because their procedures were aborted intraoperatively, one for inability to dock the working channel into or around the neuroforamen without compressing the exiting nerve and the other for superficial bleeding. Preoperative and postoperative visual analog scale (VAS) pain scores from the patients’ preoperative and postoperative pain clinic appointments were obtained from the electronic health records system to assess the intervention as a pain relief strategy. Statistical analysis was performed on Microsoft Excel for calculations of mean and standard deviations.

Surgical technique

During an ESS, the neural structures are accessed either via the neuroforamen or via an interlaminar approach. The neuroforamen is formed anteriorly by the vertebral body and intervertebral disc, posteriorly by the superior articulating process and inferior articulating process, and superiorly and inferiorly by the pedicles. This foramen is the site where the nerve root exits the spinal canal. Common pathologies leading to stenosis in the foramen and the lateral recess zone include facet hypertrophy, disc bulge/herniation, ligamentum flavum hypertrophy, facet cyst, epidural fibrosis/scarring after previous spine surgery, and degenerative changes of age. Each of these pathologies can be targeted and resected by ESS. Transforaminal endoscopy via Kambin’s triangle is the simpler approach to the neural structures of the spinal canal. Kambin’s triangle is bordered anteriorly by the exiting nerve root, inferiorly by the endplate of the proximal vertebral body, medially by the traversing nerve root and dural sac, and posteriorly by the proximal articular process [5]. Kambin’s triangle is considered a safe space devoid of vascular and neural structures where endoscopic instruments can be docked [5]. The interlaminar window can also be used for endoscopic access to the spinal canal and frequently needs facet and/or lamina resection with burrs and rongeurs to achieve optimal exposure, especially above the L5-S1 level. Interlaminar endoscopy is like an "eye inside the spine" and gives better visualization of neural structures. The working channel can be rotated and used as a joystick to elevate the nerve root or dura and look underneath the neural structures to access the disc.

**Figure 1:**
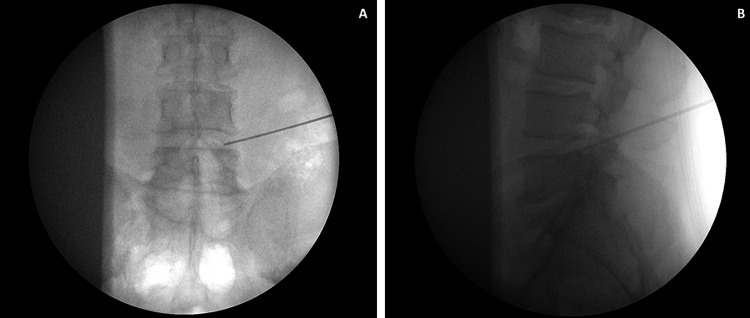
Fluoroscopic image of the needle entering the neuroforamen via Kambin’s triangle (A) The anteroposterior view. (B) The lateral view.

**Figure 2:**
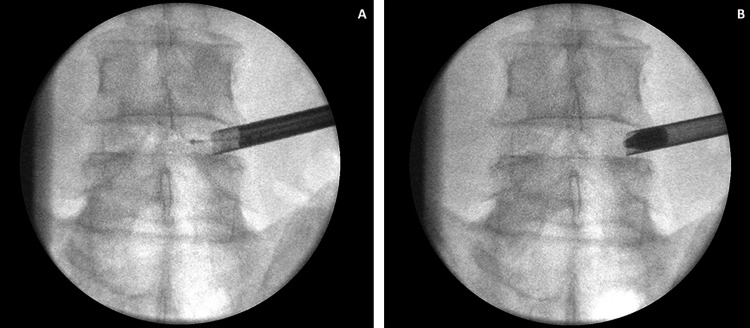
Fluoroscopic images of the working tube and tools in the spinal canal in the anteroposterior view (A) The working tube with the endoscope inside it at the right L4-5 neuroforamen. The bipolar radiofrequency electrocautery tip is coming out of the endoscope and going into the spinal canal. (B) The working tube with a blunt "pencil tip" is a dilator placed inside to get access to the right L4-5 neuroforamen.

Transforaminal ESS can be performed under local anesthesia with mild intravenous sedation, which allows the patient to communicate discomfort, as well as follow commands, thereby improving the safety profile of the procedure and guiding the end point of surgery. Alternatively, the procedure can be performed under general anesthesia with neuromonitoring using sensory evoked potentials and/or motor evoked potentials. The procedure can be performed as "inside out" or "outside in" depending on entry to the intervertebral disc, as the starting point in the "inside out" approach. Our preferred approach is "outside in": first, a needle is introduced to target the inferior pedicle under fluoroscopic guidance (Figure 1). This target provides a bony landmark adjacent to the neural foramen and exiting nerve root, which can be safely approached without harming the nerve root or advancing into the epidural space. Once needle placement is satisfactory, a guidewire is placed through the needle, and the needle is removed. Next, following a skin incision, serial dilators and trephines are placed prior to placement of a 7.2-mm beveled working tube, through which a 6.3-mm spinal endoscope is placed. Figure 2A and Figure 2B illustrate the working tube and tools in the spinal canal. To improve visualization, continuous cold saline irrigation is maintained throughout the procedure via the endoscope. Various tools can be used to remove target structures, including Kerrison’s rongeur, punch, pituitary forceps, and bipolar radiofrequency cautery. The traversing nerve root and exiting nerve root should be visualized during the procedure to free them from any compressive pathologies. If performed under mild intravenous sedation, patients often report resolution of symptoms in the operating room during or immediately after the procedure. Patients are discharged home from the recovery area on the day of the procedure.

**Figure 3:**
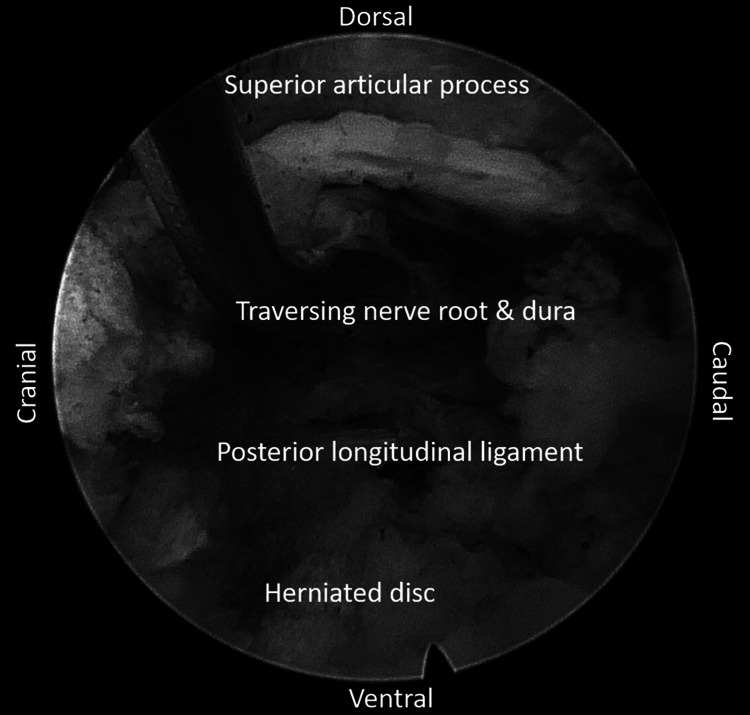
Endoscopic image of the transforaminal approach to the epidural space

Here, patients were placed on the fluoroscopic operating table in the prone position. Following “time out,” placement of monitors, surgical preparation, and draping was completed. All cases were performed under local anesthesia with intravenous sedation. The skin entry point was identified, and after injecting skin locally, a 16-gauge needle was driven targeting the inferior pedicle. A guidewire was placed through the needle and the needle was removed. Serially larger dilators and trephines for foraminotomy were inserted over the guidewire under fluoroscopic guidance. Finally, a 7.2-mm beveled working tube was placed, through which a 6.3-mm spinal endoscope was inserted. While maintaining continuous cold saline irrigation, endoscopic instruments were used to dissect/debulk the pertinent structures and complete the decompression. An anatomical image as seen through the endoscope is presented in Figure 3. Once adequate foraminal, disc, and nerve decompression were achieved and adequate hemostasis was ensured, the instruments were removed, and the incision site was closed with one absorbable suture.

## Results

Outcome data from the 20 cases were collected (Table 1). The majority of cases were performed at L4-5 (n = 14), followed by an equal number (n = 3) of cases at L3-4 and L5-S1.

**Table 1:** Demographics BMI: body mass index; SD: standard deviation

Demographic	Value
Age, years	54 (16.6), mean (SD)
BMI, kg/m^2^	40 (7.1), mean (SD)
Gender	Number of patients (N = 20)
Female	15
Male	5
Vertebral levels treated	Number of patients (N = 20)
L3–4	3
L4–5	14
L5–S1	3

Preoperatively, patients reported an average (mean) typical pain score of 7.2 (SD = 1.4) and an average (mean) maximum pain score of 8.7 (SD = 1.2) on a 10-point VAS scale (Table 2). Postoperatively, patients reported an average (mean) percent pain improvement of 82% (SD = 31) with an average (mean) postoperative typical pain score of 1.8 (SD = 2.8) and an average (mean) maximum pain score of 2.8 (SD = 3.8). The average (mean) change in pain score was -5.4 (SD = 2.8) for typical pain score and -5.9 (3.6) for maximum pain score. The average (mean) opioid dose was 18 morphine milligram equivalents (SD = 26) preoperatively and 14 morphine milligram equivalents (SD = 22) postoperatively at day three on the first follow-up.

**Table 2:** Preoperative and postoperative pain scores MMEs: morphine milligram equivalents; VAS: visual analog scale

Pain period	Mean (SD)
Preoperative	
Typical pain score (VAS)	7.2 (1.4)
Maximum pain score (VAS)	8.7 (1.2)
Opioid dose (MMEs)	18 (26)
Postoperative	
Pain improvement	82% (31)
Typical pain score (VAS)	1.8 (2.8)
Maximum pain score (VAS)	2.8 (3.8)
Change in typical pain score	−5.4 (2.8)
Change in maximum pain score	−5.9 (3.6)
Opioid dose (MMEs)	14 (22)

**Figure 4:**
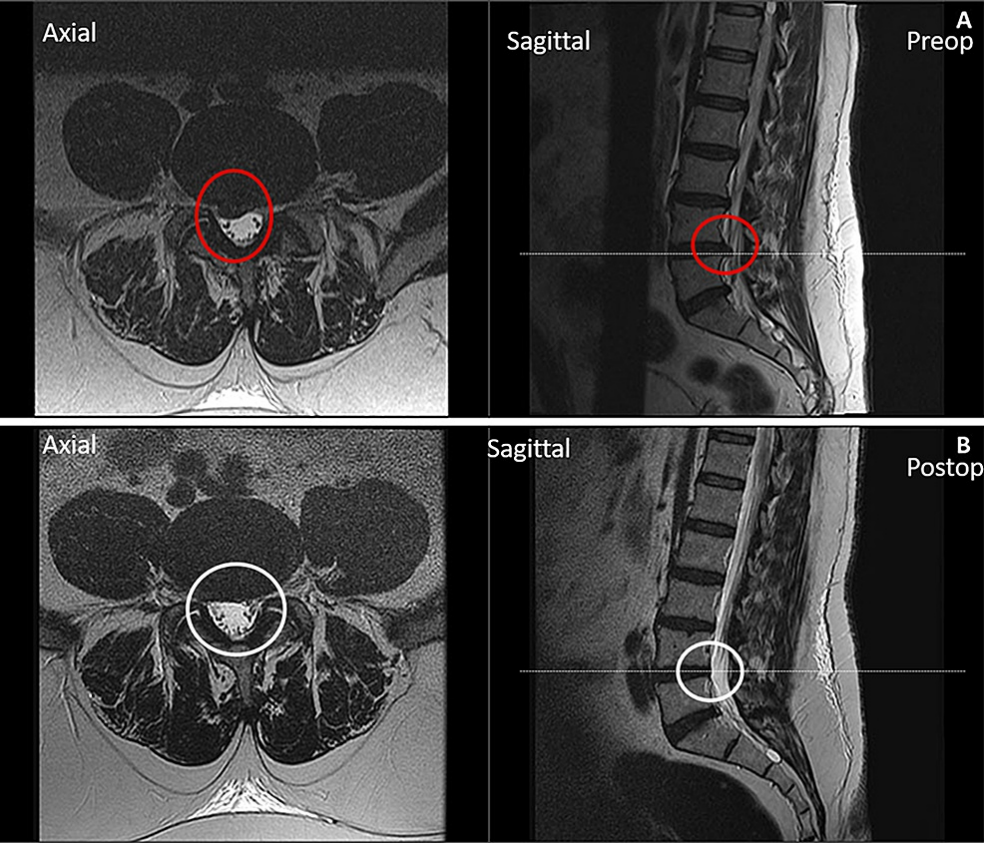
Magnetic resonance imaging (MRI) of the lumbar spine (A) The preoperative MRI axial (left) image of the lumbar spine at the L4-5 level, as indicated by the dashed line on the sagittal (right) image. The red circles highlight the pathology, namely a left-sided disc protrusion with impingement of the thecal sac. (B) The postoperative MRI axial (left) image of the lumbar spine at the L4-5 level is indicated by the dashed line on the sagittal (right) image. The white circles reflect the surgical correction of the pathology presented in panel A.

One example highlights the extent of herniated disc resection, evident in changes in imaging. Preoperative magnetic resonance imaging revealed a prominent right-sided disc herniation at L4-5 (Figure 4A), which was completely resolved in the postoperative imaging shown in Figure 4B, without any residual scarring or postsurgical changes seen in a typical open/microscopic surgery.

## Discussion

First surfacing in the late 1980s, the full endoscopic decompression technique for lumbar pathology as a minimally invasive option has evolved into a standard of care at some institutions because of its demonstrated efficacy and safety [6]. Over the years, this technique has become further refined with the addition of continuous irrigation, optimization of approach angle, and instrument development [7]. These developments improve visualization and bone resection, allowing for the safe treatment of lumbar spinal stenosis under the full endoscopic technique with similar effectiveness as open techniques [7]. Conventional decompression with laminectomy or extensive resection can lead to a variety of undesirable outcomes, including increased blood loss requiring transfusion, complications related to general anesthesia, and hospital stay. The five-year outcomes of conventional open surgical decompression have demonstrated several complications, including deterioration of function, infection, and recurrence of symptoms [8]. In addition, this operation can lead to epidural scarring, which can become symptomatic and complicate future revisions [9]. Another undesirable outcome could be tethering of the cauda equina caused by connections forming between the epidural space and paravertebral musculature postoperatively. Finally, open resection carries a risk of postoperative intersegmental instability if aggressive bone resection is required [10].

Full endoscopic decompression can provide reduced tissue trauma, preserved stability, reduced rehabilitation time, and minimal scarring while maintaining equal efficacy as more established procedures such as direct and microscopic decompression [11]. ESS can be more cost-effective due to reduced operating time and hospital stay. In fact, each of the minimally invasive endoscopic decompression procedures presented was performed safely under monitored anesthesia care in the outpatient setting.

Further advantages for the surgery include continuous, direct illumination and visualization of the target anatomy. Additionally, the operator is able to achieve hemostasis and minimal tissue disruption under continuous irrigation and drainage [12]. Beyond the advantages to the operator, perhaps most significant are the benefits to the patient. The literature has demonstrated significantly decreased pain medication requirement and operative complications in several studies when a full endoscopic technique is used [13].

The statistical significance of our case series of 20 patients is that it reinforces the benefits of transforaminal endoscopic decompression in reducing pain and opioid consumption in the immediate postoperative period, for appropriately selected lumbar spine pathology. There are some limitations in our retrospective study: transforaminal endoscopy represents a relatively small sample size of our endoscopic spine surgery patients since the majority of them undergo an interlaminar endoscopic approach. This case series also did not include a randomized design and had a limited follow-up window. We are in the process of conducting a much larger prospective study on all our endoscopic spine surgery patients, which will likely validate the finding of our small retrospective case series.

## Conclusions

This case series demonstrates that ESS can benefit patients by improving their pain and that it decreases analgesic requirements at postoperative day number three. Future studies can be aimed at including all our endoscopic surgery patients and can include long-term follow-up and outcome tracking, including any symptom recurrence in patients receiving the full endoscopic technique. In conclusion, endoscopic spine decompression has proved to be a beneficial technique for the patient and an advanced skill set for the operator when performed by properly trained pain management physicians.
